# An Effective Self-Configurable Ransomware Prevention Technique for IoMT

**DOI:** 10.3390/s22218516

**Published:** 2022-11-04

**Authors:** Usman Tariq, Imdad Ullah, Mohammed Yousuf Uddin, Se Jin Kwon

**Affiliations:** 1 Department of Management Information Systems, CoBA, Prince Sattam bin Abdulaziz University, Al-Khraj 16278, Saudi Arabia; 2 College of Computer Engineering and Sciences, Prince Sattam bin Abdulaziz University, Al-Khraj 16278, Saudi Arabia; 3 Department of AI Software, Kangwon National University, Samcheok 25913, Korea

**Keywords:** Internet of Medical Things (IoMT), ransomware, Cyber-Security, Tizen OS, Cuckoo Sandbox

## Abstract

Remote healthcare systems and applications are being enabled via the Internet of Medical Things (IoMT), which is an automated system that facilitates the critical and emergency healthcare services in urban areas, in addition to, bridges the isolated rural communities for various healthcare services. Researchers and developers are, to date, considering the majority of the technological aspects and critical issues around the IoMT, e.g., security vulnerabilities and other cybercrimes. One of such major challenges IoMT has to face is widespread ransomware attacks; a malicious malware that encrypts the patients’ critical data, restricts access to IoMT devices or entirely disable IoMT devices, or uses several combinations to compromise the overall system functionality, mainly for ransom. These ransomware attacks would have several devastating consequences, such as loss of life-threatening data and system functionality, ceasing emergency and life-saving services, wastage of several vital resources etc. This paper presents a ransomware analysis and identification architecture with the objective to detect and validate the ransomware attacks and to evaluate its accuracy using a comprehensive verification process. We first develop a comprehensive experimental environment, to simulate a real-time IoMT network, for experimenting various types of ransomware attacks. Following, we construct a comprehensive set of ransomware attacks and analyze their effects over an IoMT network devices. Furthermore, we develop an effective detection filter for detecting various ransomware attacks (e.g., static and dynamic attacks) and evaluate the degree of damages caused to the IoMT network devices. In addition, we develop a defense system to block the ransomware attacks and notify the backend control system. To evaluate the effectiveness of the proposed framework, we experimented our architecture with 194 various samples of malware and 46 variants, with a duration of sixty minutes for each sample, and thoroughly examined the network traffic data for malicious behaviors. The evaluation results show more than 95% of accuracy of detecting various ransomware attacks.

## 1. Introduction

The Internet of Things (IoT) has successfully managed the growing needs of societies by improving quality of life and has efficiently deployed a range of services, such as smart cities, agriculture, healthcare and emergency health services etc. Among the various applications of IoT, the implementation of the e-health Internet of Medical Things (IoMT) is playing a vital role in the healthcare industry with the aim to gain efficient health access, improve the hospital healthcare quality, and improve the productivity of medical equipment [1,2]. To enable IoMT, a diverse range of sensor devices are connected together with IoT-enabled technologies in order to deliver reliable healthcare services e.g., distantly monitoring patients [3,4] and effectively communicating and advising [5]. Researchers have been working to create healthcare frameworks that link medical equipment to a variety of healthcare services in a way that is accurate, reliable, and most importantly secure, all in an effort to enhance the healthcare industry as a whole via IoMT [6]. Various types of security attacks over IoT [7], especially controlling the medical devices in an IoMT e.g., for ransomware, have been affecting the communication of IoMT devices that could threaten the patients’ lives and disable the entire healthcare system.

The ransomware attacks are rapidly growing and are threats to individuals, businesses, and government and private sectors, where the attackers infect their deployed infrastructure and demand large amounts of ransom [8,9,10]. Due to an excess of such attacks, newer versions of the ransomware attacks appear frequently and have been responsible for millions of dollars of losses annually [11]. Unlike conventional malware attacks, which often target a single layer of an IoT network, ransomware may spread across the network, wreaking havoc on several levels before ultimately compromising the network’s security in the process e.g., integrity, confidentiality etc. This has a disastrous effect on essential real-time systems such as IoMT since it leads to money losses and vital information leaks. A ransomware attack not only takes control of the network infrastructure of IoT network, it can also take control of the user data, resulting in limited or no access to the service data and operations. If the victim refuses to pay, the ransomware attack may result in extended period of attack, increased demand, or the data may be deleted by the attacker [11].

The ransomware attacks can be divided into three categories: Crypto ransomware [12] where the attackers use the encryption and decryption algorithm (i.e., public-private key) to encrypt the data and files, hold the decryption key, and demand for ransomware. The decryption key is handed over once the victims pay the ransom amount. In case of the IoT networks (e.g., IoMT), the crypto ransomware is launched over the backend application servers instead of IoT devices since the sensor devices do not contain large amounts of data. Locker ransomware blocks user access to IoT devices, disables IoT sensor devices, and controls system performance. In addition, this attack can be combined with the DDoS attacks to disable user interface with the IoT devices or inactivate the sensor nodes, thus denying legitimate user access. The Hybrid ransomware encrypts the important devices’ data (i.e., IoMT devices) and uses locking mechanism, which is severely devastating since both the data and overall system functionality is compromised.

In this paper, we develop a ransomware analysis and identification architecture that detects ransomware attacks and ensures their validity and accuracy using a comprehensive verification process, by experimenting with various types of malwares. We took ten different malwares, e.g., GPcode, Filecoder, etc., and experimented on their various samples (respectively with an Avg. and St. Dev. of 21 and 18 samples) and variants (respectively with an Avg. and St. Dev. of five and two malware variants). Hence, we evaluated our ransomware analysis and identification architecture with a total of 194 various samples of malware and a total of 46 variants. For each of the selected malwares, we considered various types of ransomware attacks e.g., ‘Encrypting Files’, ‘Deleting Files’, ‘Stealing Files’. We conducted our experiments using a Cuckoo Sandbox [13] that helps analyze the network traffic data, specifically encrypted with Secure Sockets Layer (SSL) and Transport Layer Security (TLS) protocols, for malicious behavior of network peers. In addition, for each of the 194 samples, we ran our experiment for a total for sixty minutes and thoroughly examined the network traffic data for malicious behaviors.

The primary goal of this malware analysis is multifold: How a malware attack interacts with file system associated with IoMT devices, the nature of attack e.g., changing metadata of a file system, to evaluate the robustness of a secure software system e.g., OpenSSL, against eavesdropping, to differentiate between static and dynamic ransomware attacks, and to verify the integrity via registry of a file system. In addition, we followed various guidelines [14,15] in order to ensure the validity and accuracy of ransomware detection analysis i.e., ensuring that the testing dataset comprises of diverse range of malwares, the malware attack is robust against different system configurations to experiment various settings and security measures, the target is within the vicinity of connected IoMT devices, in addition to guaranteeing that the detection filter has access to optimize various signatures of source files.

During each trial session, the system log was copied to a parallel storage location, where the responses were subsequently aggregated, evaluated, and audited. This was done in order to improve anomaly detection and make our detection process as effective as possible. Throughout this process of detection and optimization process, we took several measures and steps: to see if the files were setup in diverse locations, separate file setup was implemented to launch a different attack, search for corrupted files, and the files are concurrently generated. After every iteration of our experimental session, we record a detection rate which has a precision that is more than 85% of the detection accuracy. Similarly, for each of the iterations, we note an average latency of malware detection was recorded nearly 0.03 and 0.025 ms, respectively for continuous and triggered scanning of malware. Furthermore, for various timestamps of the experiments, we found an increase of malware detection of our proposed detection model. We argue that this performance is satisfactory: it demonstrates the system’s consistency once an anomaly is initiated by the ransomware.

Our main contributions are:

- Developed a ransomware analysis and identification architecture that detects ransomware attacks within an IoMT network
- Developed a comprehensive experimental environment to simulate a real-time IoMT network
- Constructed a comprehensive set of ransomware attacks and analyze their effects over an IoMT network devices
- Developed an effective Detection Filter for successfully detecting various ransomware attacks and evaluating the degree of damages caused to the IoMT network devices
- Developed a defense system to block the ransomware attacks and notify.

We organize the paper in the following sections: Section 2 presents the related work. The proposed methodology along with the experimental setup is presented in Section 3. Section 4 presents the performance analysis of our proposed ransomware analysis and identification architecture. Section 5 presents experimental results; we conclude in Section 6.

## 2. Related Work

Urooj et al. studied the dynamic analysis for ransomware detection across multiple platforms, dataset collection and application of machine learning, deep learning and hybrid approaches in dynamic analysis of ransomware detection. This study also proposes future research directions. This study listed twenty-three windows platform datasets, two Android, four cloud and IoT datasets and two network based datasets used in dynamic analysis. Dynamic analysis executes the malicious code in a controlled environment to capture the ransomware behavior. Pre-encryption detection studies are very limited according to this study. This study addressed the open issues in ransomware detection for future research: real-time detection, time-complexity, implementation on low specification hardware, evasion and obfuscation-tolerant systems. This article provides comprehensive study on dynamic analysis for ransomware detection [16].

Humayun et al. provided a wide-ranging analysis on evolution, prevention and mitigation of ransomware in IoT. This study reported the current research directions for ransomware during the period of 2014 to 2018, the penetration in different countries and statistical report on ransomware attacks. Further ransomware propagation through various sources and most affected sectors. Healthcare sector is top most priority for the attackers including government and education sectors. This survey revels more priority should be given in studying prevention and mitigation of ransomware attacks on IoT enabled environments [17].

Alrawashdeh et al. offered a rapid method of ransomware detection using Memory-Assisted-Stochastic-Dynamic-Fixed-Point arithmetic using a four-layer Deep Belief Network (DBN) structure. In this approach, efficient cross-correlation for the stochastic computation in FPGA is produced by storing random bit-streams in memory. The memory technique for the Deep Belief Network (DBN) is trained with stochastic computation with dynamic fixed-point arithmetic. Precision rate 91% and detection speed of 0.006 ms. is reported. This approach improves ransomware detection of ransomware in Internet of Things (IoTs) [18].

Homayoun et al. presented ’Deep Ransomware Threat Hunting and Intelligence System (DRTHIS)’ to detect ransomware and its family using Long Short-Term Memory (LSTM) and Convolutional Neural Network (CNN) deep learning techniques, for classification using the softmax algorithm. Training dataset includes 220 Locky, 220 Cerber and 220 TeslaCrypt ransomware samples, and 219 benign samples. Test results achieve F-measure of 99.6% with a true positive rate of 97.2%. This model is even tested with previously untrained variants of ransomware families CryptoWall, TorrentLocker and Sage. Results for new ransomware detection rate are 99% of CryptoWall, 75% of TorrentLocker and 92% of Sage. This study focused on fog layer nodes, which holds sensitive data [19].

Azmoodeh et al. proposed a method in which energy consumption patterns are monitored to detect the ransomware. Energy consumption rate of different process is used to classify ransomware from non-malicious applications on Android devices. PowerTutor is used monitor power consumption of all running processes in 500 ms intervals. Ransomware samples from VirusTotal, Intelligence API are applied with active Command and Control (C2). This study demonstrated proposed approach outperforms K-Nearest Neighbors, Neural Networks, Support Vector Machine and Random Forest, in detection rate, recall rate, precision rate and F-measure [20].

Hatzivasilis et al. studied the security and privacy requirements and best practices, which can be adopted to safeguard the users and stakeholders along with IoMT system. Authors consider this study could be used as practical guide for developing IoMT application. Security areas considered by most of the popular vendors are devices, security at connectivity and cloud levels and security aspects studied here are confidentiality, integrity, and availability. Different protection mechanisms discussed in this study. Privacy of personal data stored in IoMT devices is equally a high priority requirement. There are established standards for privacy protection of Personal Identifiable Information (PII), similar to the ISO/IEC standards 27018 and 29100 and regulation efforts such as the General Data Protection Regulation of European Union Regulation (EC) 2016/679 was established. Data is categorized into personal sensitive, sensitive and statistical. Highest privacy protection is given to personal sensitive data followed by the two others. Three aspects of privacy studied in this paper are data-collection, data-access, and data-usage. Protection mechanisms for these aspects are discussed and described [21].

Tervoort et al. reviewed options for mitigating cybersecurity risks from legacy medical device software. This study found eighteen solutions for intrusion detection and prevention, communication tunneling insecure wireless communications. This scoping review focus on vulnerabilities in medical devices due to lack of security features, legacy operating systems, unsupported software and inability to apply patches. To deal with the security issues of medical devices that run legacy software a scoping review using a bidirectional citation searching method. Search started with three relevant studies and discovered 121 articles cited by these three studies and 725 articles cite these studies. Results classified based on four criteria: (a) Application area (b) Risk type (c) Solution type and (d) Method of analysis. The authors found 18 studies that address the risk caused by legacy software in medical devices [22].

Fernandez et al. [23] proposed an intelligent and automatic solution to detection, classification and mitigation of ransomware attacks in integrated clinical environments (ICE++). ICE++ combines the mobile edge computing (MEC), software defined networking (SDN), and Network Function Virtualization (NFV) provides adjustable, cost-effective, and self-regulating administration of security system to mitigating the ransomware attacks in ICE. The proposed system consists of four modules: the monitoring module collects the network traffic from medical devices and generates feature vectors, the offline model generation module receives feature vectors generated training dataset and selects a classification algorithm to train a model and the analyzer module accepts the qualified ML models from and, in real time, the current feature vectors thereafter detects the anomalies and labels the traffic as ransomware or benign. Finally, the decision and reaction module assesses the risk of having a functioning ransomware attack. This system uses One Class SVM for anomaly detection and Naive Bayes for probability valuation of the association of the new models to the class with the best analogous traffic pattern. Performance of the proposed model is significant: it achieved 92.32% of accuracy and 99.97% of precision in abnormality recognition; however Naive Bayes achieved a 99.99% of classification precision.

Baek et al. [24] suggested a two-step hybrid malware detection system (2-MaD) to guard IoT nodes against obscured malware in a smart city environment. The 2-MaD program was split into two steps. First, in which the detection of malicious software is carried out using static analysis, and second, where the detection of malware is carried out using a dynamic scan. The correctness of the false negative rate (FNR) was used as a performance indicator. The performance assessment for 2-MaD exhibited that the malware discovery precision was 94.46%, which exceeded detection based on static analysis. The shortcoming of the projected scheme is that it failed to emphasized on involved application interface calls, instruction trace logs and track the registry changes.

Damien et al. [25] offered a feature section architecture (FeSA) that aims to discover a set of ransomware features that helped to develop the sustainability of the applied machine learning classifier. The functional technique was equated to other systems such as evolutionary search, harmony search, etc. in order to measure the ransomware exposure degree, recall, false negative and accuracy. The projected mechanism failed to highlight the exploitation impact of victim node’s boot record. Moreover, the ransomware identification and prevention paradigm was not thoroughly investigated at gateway that is prime spot for effective anomaly detection and response.

Farnoush et al. [26] identified ransomware only consuming the headers of the executable file by creating and mapping feature vector graph using ‘Power Iteration’ technique. For evaluation purpose, three datasets were compiled. The first data set contained 12,000 portable executables whereas the second dataset was based on 2000 executables. The third data set was assembled with malwares such as Wannacry, Cryptowall, etc. with file sizes ranging from 1kb to 26mb. The core benefits of the offered technique are satisfactory computational convolution and satisfactory ransomware discovery rates.

Zahoora et al. [27] proposed a novel Deep Contractive Autoencoder based Attribute Learning (DCAE-ZSL) system. The projected method was able to efficiently discover code insertion that can study the semantic depiction of zero-day attacks in an unsubstantiated style. To examine the method effectiveness the applied dataset comprises of 582 ransomware and 942 normal software samples based on office productivity apps, mobile gaming APK and Microsoft Windows OS compatible widgets. Scheme accomplished a substantial conciliation between false positive and false negative as associated to the shallow baseline prototypes. DCAE-ZSL is only effective on executable files, and does not show promising outcome for diverse family of ransomware anomalies.

Manabu et al. [28] presented an open data set about hypervisor-based ransomware storage intake behaviors. The dataset contains entree configurations of ransomware options that considers variable OS versions and encryption applied methodologies as a benchmarking criterion for sample segmentation. Dataset validity was examined by applying feature engineering, and confusion matrices, which allowed to gain five-dimensional data vectors. The main limitation of focused research is that the dataset was compiled and assembled in consideration with an obsolete operating system (i.e., Windows 7) and will not be helpful to train system against emerging ransomware tools.

The Industrial Control System (ICS) links the virtual and natural worlds with various physical components, such as sensors and controllers. The present installation of the ICS is mostly housed in what is known as the “plant”, which is a technical name for the facility’s physical infrastructure. Inputs, outputs, and logic are the three components that make up control systems in IoT enabled facilities. Control inputs transmit plant status. Zhang et al. [29] claims control logic is PLC software that can repeatedly receive, calculate, and deliver control signals. This article introduces ICS-ARC, an innovative ransomware attack mechanism that can automatically construct network packets unique to specific control logic. The approach is presented in this article. A cyberattack using ransomware is carried out by ICS-ARC in a four-step process. Both an Arduino with pre-installed OpenPLC and a separate tap water treatment system were constructed by the authors so that they could test the systems’ ability to exploit ICS-ARC vulnerabilities. The findings of the demonstrated operational anomaly reveals that ICS-ARC substantially increases the fault tolerance of the attempt while simultaneously decreasing the cost of the attack.

The extensive implementation of various information technology platforms across a wide range of enterprise sectors is what is meant to be referred to as “digitization”. The process of transformation often makes use of several sorts of systems, such as software platforms, computer networks, and other types of network infrastructure. To identify phishing simulation research methods, technologies and research gaps in practically evaluated literature, a comprehensive review was carried out by Yeng et al. [30]. Phishing serves as the most prevalent ransomware assault because it exploits the weakest link. An SMS-based spoofing scenario research was conducted on Ghanaian medical practitioners using state-of-the-art and quantitative techniques. For evaluation, the following roadmap was adopted: (a) create content that is intentionally misleading, distribute a leaked document to the authorities, and get approval in advance; (b) examine the safety of the individuals involved as well as the security of their private details; and (c) claim about doing a debriefing, getting post-consent feedback, and protecting your data. Research concludes that 61 percent of recruited healthcare professionals were vulnerable.

Alqahtani et al. [12], offered a survey that is dedicated to studying and assessing the state-of-the-art in ransomware detection and prevention in the interest of aiding the scientists that attempts to disrupt this extremely significant and rising malware issue. The emphasis is on cryptographic ransomware since it is the kind that is the most common, damaging, and difficult to deal with. This article reviews the methods used for ransomware identification modeling to provide suggestions for the focus and orientation of research directions. Furthermore, concerns about ransomware early detection were addressed in this article. Approaches pertinent to the various detection method stages have been investigated. Authors have extensively commented on the current efforts that aim to enhance feature extraction, selection, and behavioral modeling. Article suggested that innovative techniques and solutions are still required, particularly those that examine combined data from various sources and systems to strengthen and enhance malevolent software’s behavioral traces.

Table 1 summarizes the purpose of various research works, characterized over various features of ransomware, e.g., objective, contagion, malevolent events and extortion, along with various tested environments and platforms i.e., PCs, mobile device, and IoT.

## 3. Proposed Method

Today, more than twelve billion devices are interlinked with each other to sense, aggregate, process, analyze and exchange valuable streams of data [31]. Each device has a unique identifier which enables the functional user to seamlessly operate and perform rightful action. Considering the actionable gains, researchers are experimenting different use cases that will enhance the productivity, efficiency, and effectiveness of healthcare infrastructure. Mobility-aware digital healthcare appliances (i.e., Internet of Medical Things (IoMT)) are helpful in ‘remote patient monitoring (RPM)’ by leveraging the connected devices equipped with IoT sensors to monitor the fitness of a person and feed the sensed data directly to ‘electronic health record (EHR)’ system. EHR is a vital decision-making tool for physicians.

IoMT sensors help caregivers to capture reliable and contextual data feeds, such as a patient’s status of his/her physiological, heart rate, glucose level in blood, oxygen saturation, etc. Automation in hospitals reduces the cost and enhances the quality of care. For hospital staff, their prime focus is to keep the patients happy and healthy. Unfortunately, security and privacy are an exploitable consideration. Connected devices, such as fitness monitors, sensors, affiliate data sources can be maliciously abused and disrupt the flow of information to/from the needed platform. One of the emerging risks is associated with ‘ransomware’ that locks the victim machine to support adversary’s illicit intentions. Malwares (such as constructor, behavior analyzers, backdoors, SQL slammers, and Crypto-Lockers) are able to deploy evasion methods, infection broadcasting, and distributed Denial of Service (DDoS) attacks. Unlike traditional malwares, ransomware target ‘Command and Control (C&C)’ servers and health monitoring interconnected devices to exhaust and lock the daily operations of an organization.

**Figure 1:**
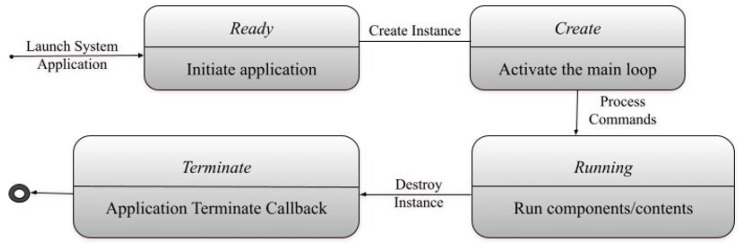
Module based application statuses.

Proposed research focuses on devices mounted with Tizen [32] operating system. Tizen was programmed in consideration with rapidly changing mobility aware sensing devices and for affiliate data-driven systems. Tizen module supports Linux-based essential libraries that are helpful for EHR databases, data parsing, connectivity-related functionalities, personal information management (PIM), etc. It sustains an applied method and its properties using cgroup (i.e., control group). The significance of the process is stimulated to lessen the likelihood that the OS event progression is killed in a low memory condition. Moreover, sensor frameworks furnish instrument proceedings to applications and platform modules. A sensor operation can be evaluated by engaging hardware or simulated sensors. Table 2 describes the application & system states as illustrated in Figure 1.

By extracting application statues, information such as Resource ID, Resource Type, URI (i.e., provides data related to Authority, application Path, system Query), Multipurpose Internet Mail Extensions (MIME) type, application & message keys (for example: app_control_data, etc.), and launch mode settings (i.e., privileges) can be revealed. In projected technique, for messaging ‘remote & local’ ports were accessible. Tizen platform secures accessible data and mounted applications by storing them in a secure repository and crypto modules.

Using the popular OpenSSL command line program, we implemented key management, data integrity, ciphering, and decoding. The following cryptographic techniques were utilized: (a) Data Encryption Standard (DES), (b) RSA (Rivest–Shamir–Adleman), and (c) a digital signature algorithm (DSA). Tizen has a built-in Content Screening and Reputation (CSR) framework that grants privilege to installed application to filter, audit and block malicious event calls. Each application is eligible to code its malware scanning criteria by (a) forming content screening context, (b) applying content scanning (i.e., memory, file, & directory), (c) detect a malware by signature verification (i.e., size, exploit path, event list & size, and device privilege policy).

Contrasting most of current malware attacks, ransomware threats are not typically considered to be furtive after the septicity level, as the entire idea of the attack is to report targets that their computing infrastructure is maliciously compromised with the aim to encrypt or destroy the data. To understand the ransomware triggering behavior of malicious software we have analyzed the following (Table 3) original and upgraded malware:

Experiments were conducted using modular Cuckoo Sandbox [33] that permits analysis of memory, behavior of malicious files and network traffic especially when the data stream is cyphered with Secure Sockets Layer (SSL)/Transport Layer Security (TLS). Each sample was rigorously examined for sixty minutes. After each session, the system was refreshed and rolled back to normal state to diminish or avoid any intrusion by secondary evaluations. The prime goal of malware analysis was:

1. To define by what means a malevolent procedure interacts with the file system after a victim IoMT device is experiencing a ransomware attack.
2. To evaluate I/O callback routines that handles the ‘feature change call’ to the file system.
3. To analyze the reliability of customized cryptosystems (i.e., OpenSSL and YACA).
4. To understand the static (code) and dynamic (behavior) of ransomware.
5. To verify the integrity of registry keys, registry process, Dynamic-link library (DLL), UNICODE strings, information about TCP connections, TCP dumps, data related to ‘file_change_monitor’, TCP port listener, and unpacked applications.

### Detection Filter

We assume that if malware is present in the system, it influences more than one application-driven data set. Whether the adversary attempt was successful or failed, the corresponding data were logged. If a violation (i.e., malicious event or behavior) is observed, the defense system will block the event and notify the base station.

(1) $$\begin{array}{cc} \; & \sum_{Executions}\left[kO\times \left(\frac{1}{mK}\;\times \;\frac{1}{pos}\right)\right]=Mo\;with\;\;\;\;\;\;\\ \; & \begin{array}{cc} kO=1\;\;\;\;\;\; & \mathrm{if}\;\mathrm{the}\;\mathrm{execution}\;\mathrm{comprehending}\;\prime H\prime \;\mathrm{failed},\;\\ kO=0\;\;\;\;\;\; & \mathrm{otherwise} \end{array} \end{array}$$

Here, ‘*kO*’

is the entropy (i.e., state of disorder), where ‘*O*’ is the unique group of samples, ‘*H*’ is the associated malicious event linked with ransomware, and ‘mK’ is the analyzed log file. As per Equation (1), the number of unsuccessful executions in which ‘*H*’ is available (*Mo*) over several executions that exist in (*M*). ‘*pos*’ is represented as a Boolean function that signifies the ‘open or false’ state of policy logic.

k=K(H,i.e.,isthemaliciousactivitylinkedtoransomware)

(2) =Mo+∑executionkO×(1mK)α×(1pos)β2M

According to the Equation (2), there is a strong chance that the fault/Trojan horse is embedded in the system routine if there are abuses signaling a similar procedure. Thus, the use of probabilistic score estimation is beneficial in the process of assigning relative ratings to all interconnected devices. For a given IoMT ad-hoc network ‘N=(H,P)’ with |L| devices, let ‘T=(tyx)’ be the parsimony model; if vertex ‘*y*’ is linked to ‘*x*’, so ‘tyx=1’, and ‘tyx=0’ otherwise. Here ‘*N*’ is the entropy rate of the ad-hoc network, and ‘P’ is the state of malware transmission matrix.

(3) $$d_{y}=\frac{1}{\exists }\sum_{x\epsilon U(y)}d_{x}=\frac{1}{\exists }\sum_{x\epsilon N}t_{yx}d_{x}$$

where ∃ refers to existential quantification that was used to identify predicate variable and U(y) is a regular dataset of related event log associated to constants ‘*y* and ∃’. Application call-related event data require a ranking matrix to identify the importance of generated data logs.

(4) $${KB}_{y}=\frac{1-A}{M}\;+a\sum_{x\epsilon H(y)}\frac{KB(x)}{outQ(x)}$$

where ‘*a*’ is the datasets, ‘H(y)’ is a set of relationship among data connections to ‘*y*’, ‘OutQ(x)’ is the outer links to external data sources available of node ‘*x*’, and ‘*M*’ is the total number of associated interlinked devices.

During experiments, we observe that during malware transaction, the short-time power consumption and frequency-domain function outcome data are drastically variable in comparison from the routine behavior. The Laplace transform function was applied to projected method to analyze continuously generated variable case data to resolve the initial value problem.

(5) Dw=∑w=0M−1dwp−x2πxwm,(0≤w≤M−1)

Equation (5) indicates the possibility to observe and evaluation of linear continuum ‘D(w)’ which indicates exposure of observed system to the malevolent entity. To match the signature, a requirement-focused characteristic database is required and was maintained. For this reason,

(6) $$A_{y}\left(f^{y}\right)=probability_{d}istribution\left(f^{y}|d^{y};\exists \right)$$

where ‘*y*’ represents the chosen preparation data section, and ‘*f*’ is the classification in which this dataset can fit in to, and Ay refers to a definite likelihood distribution of ‘*f*’.

(7) $$measured\;variable\;\left(\theta \right)={max}_{\theta }\sum_{y}\sum_{f^{y}}A_{y(f^{y})}\log\frac{probability(d^{y},f^{y};\exists )}{A_{y}\left(f^{y}\right)}\;$$

For analysis, we collected, processed, and analyzed data captured in context of CPU energy consumption when specific health monitoring applications were active. In healthcare facility, EHR was enriched by real-time sensor feeds (e.g., biosensor data flow, images, accelerometer, temperature, and pressure). Sampling frequency for data aggregation of required data to identify ransomware was 200 ms per sample. Nominated flags to identify the ransomware existence in the host device are identified in Table 4.

The ransomware executable packages itself with numerous executables that are prerequisites for it to achieve its operational requirements. During the experiment, the projected ransomware used SHA-256 to partially encrypt each file separately, change and rename the file type extension identifier, change the wallpaper on the device, programmatically avoid the caching suspension, and skew the examination of the encrypted binary file.

Here, we assumed that skewness is a degree of regularity in a dissemination. Momental skewness (*E*) can be evaluated as:

(8) αu=12E1=μ3/3σ3

where σ is the regular eccentricity, μ is the mean and ‘*E*’ is referred as skewness. Since the suggested experimental system could provide a mean and a standard deviation, the skewness of the data set at the time of evaluation could be calculated. In consideration of Equation (8),

(9) $$E_{3}=\frac{\sum_{y}^{M}{\left(D_{y}-\bar{D}\right)}^{3}}{\left(M-1\right)\times \sigma^{3}}$$

where ‘*D*’ is random variable, and ‘$\bar{D}$’ is the mean of distribution.

In general, proposed technique considers an unfamiliar executable (i.e., mentioned in Table 3 and Table 4) as apprehensive, for the reason that these kinds of process complication practices are commonly applied by malwares.

## 4. Performance Analysis

**Figure 2:**
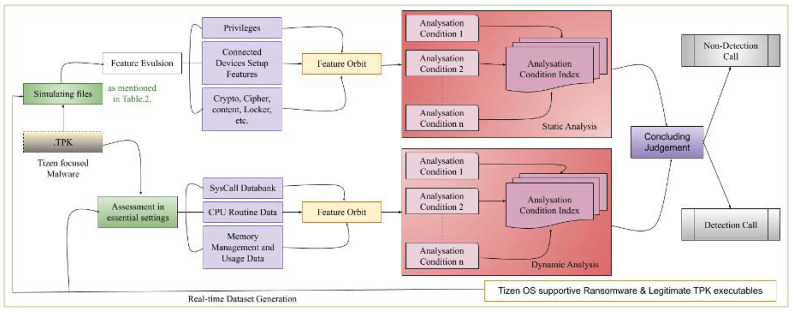
Ransomware analysis and identification architecture (i.e., before Denial-o-Service).

In consideration with Tizen ransomware attack phases, the malware identification, tolerance, and countermeasure mechanisms were coded for characterization and evolution purpose. Various configuration settings for the experimental evaluations are given in Table 5. Dataset (i.e., Table 3) analysis was conducted for the following system configurations:

Before performing feature assortment, we have prepared our data set by eradicating duplicate features. For applied architecture mentioned in Figure 2, dataset weight was approximately 149 GB, which contains source files, data files, database backups, xml supported multimedia files, TPK, etc. To ensure validity and accuracy of ransomware detection analysis, we followed pre-declared guidelines, such as:

1. Verification of testing dataset by ensuring if it contains dissimilar malwares,
2. Malware is effective in different system configurations,
3. Intended target is linked with Prince Sattam University’s internal or external network devices, and
4. Detection filter is capable of editing system ‘access control policy’ source files in order to tune and optimize certain signature or behavior.

The following factors are maintained constant during the entire experiment.

1. The experimental configuration on which the test is performed.
2. The ransomware agent used to attack the target system.
3. The mechanism that detects ransomware.
4. The timing mechanism for system shutdowns.

To apply weights to each evaluated feature, we used Logistic Regression (LR) model. LR provides an effective understanding of relationship among dissimilar dataset variables that helped project system to decide whether certain system call (e.g., ‘feature change call’) is malicious or legitimate.

(10) $$\rho \left(d\right)=\frac{1}{1+p^{-d}},\;\;d=\beta_{0}+\beta_{1}d_{1}+\ldots +\beta_{m}d_{n}$$

where $\beta_{m}$ the erudite ratio for feature $d_{n}$. This ratio is erudite through repetitions in order to diminish the inaccuracy between the anticipated values and the definite values.

## 5. Experimental Results

**Figure 3:**
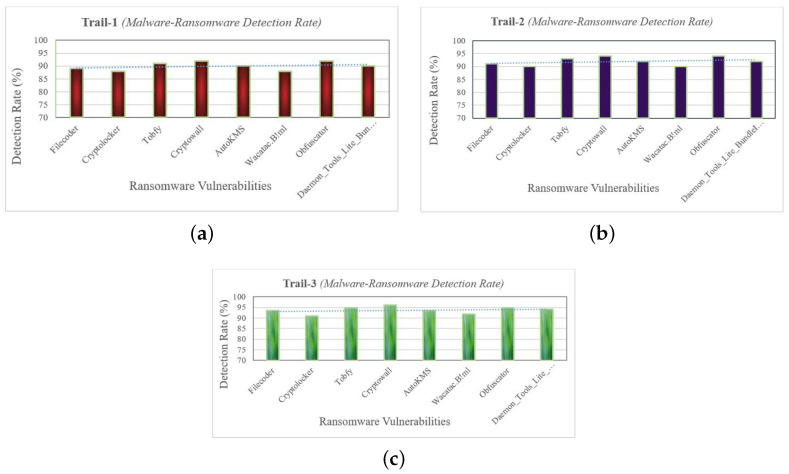
Ransomware detection rate (**a**) Trial 1, (**b**) Trial 2, (**c**) Trial 3.

Malware implies the use of common techniques, as illustrated in Table 3. To determine how the malware binary is generated and responded, three experimental trials were carried out. For each session, 60% of the sample set was used for training and 40% for testing the intended method. The results of each experimental session are set out in Figure 3. The primary aims to conduct detection trails were (a) to scan folders using signature-based detection to identify files and patterns related with ransomware, and (b) by using rule-based analytic, find ransomware strains that have not been seen before. After each session, the system response log was aggregated, analyzed, and audited to enhance the anomaly detection system. Analyzed ransomware accesses a variety of directories to establish an appropriate environment for ciphering.

**Figure 4:**
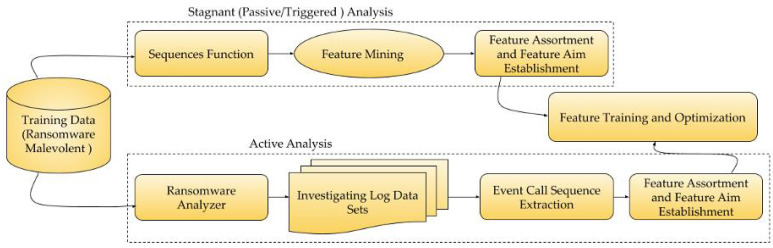
Ransomware’s Feature detection and Optimization Process.

Figure 4 (i.e., extension of Figure 2) focuses on the feature extraction that is required to identify and optimize vulnerability detection process. The following characteristics were exclusively (but not limited to) evaluated for each iteration:

1. If directories/files are set up in various locations?
2. If the created folders begin with a distinctive character?
3. If a separate filing setup is implemented?
4. Do files that are created have understandable names?
5. Are various file formats taken into consideration?
6. Whether the files are unusable (corrupted) files?
7. If more than 50 files are generated concurrently?
8. If an alert is triggered when detection log files are viewed?

**Figure 5:**
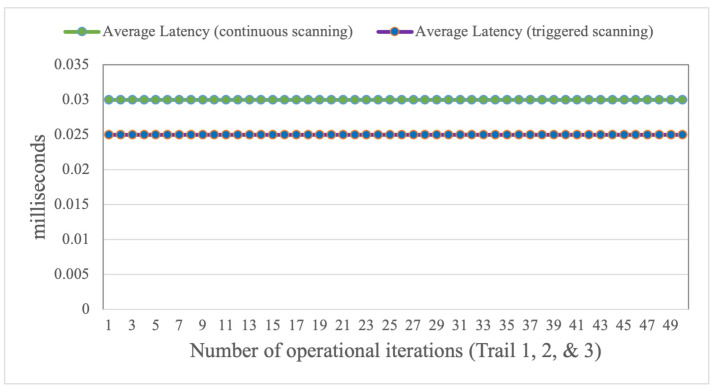
Average Latency impact on Tizen mounted devices.

Figure 5 indicates the mean latency, which is logged and compared after the detection technique has been carried out to determine whether the performance of other transactions is stimulated by the applied detection approach. In terms of performance measurement after registering the aforementioned activities, devices were periodically exposed to predefined vulnerabilities. Once the infection scenario begins, the start and end times of the events are recorded to calculate the average overhead.

**Figure 6:**
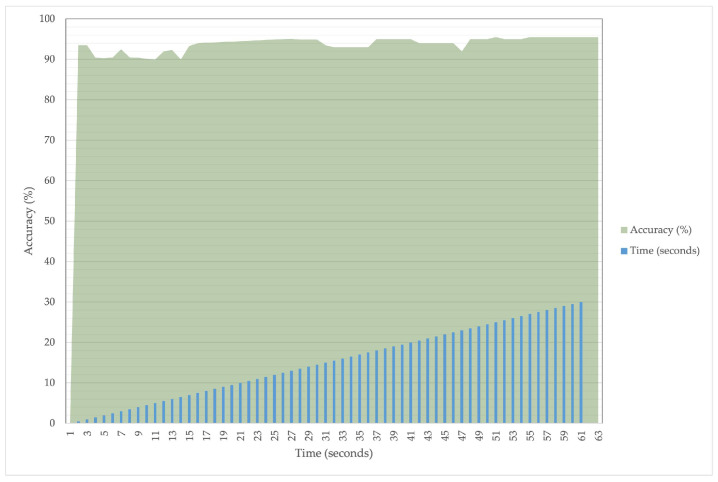
Early Detection Accuracy.

Figure 6 shows how the model performs on different timestamps. The output is not constant every second, but it is usually high because it is equal to or over 90 percent. For quick malware recognition, this performance is satisfactory because it demonstrates consistency once anomaly is initiated by the ransomware.

**Figure 7:**
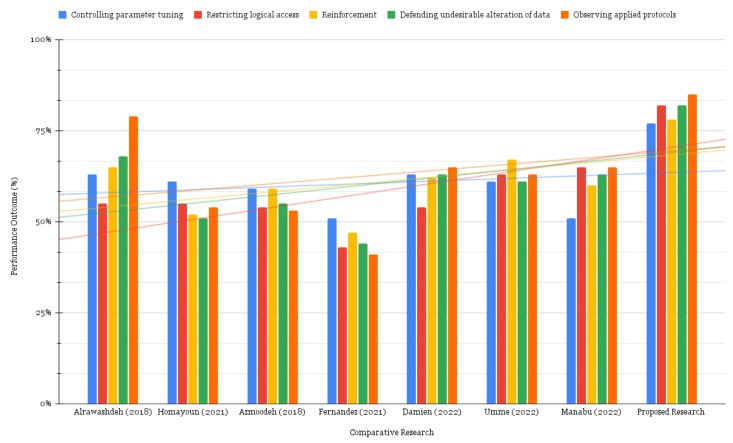
Comparative Analysis [18,19,20,24,25,27,28].

Figure 7 exhibits a comparison of the suggested technique with that of previously published studies. It is apparent that there are currently no solutions that can suit all the requirements that have been described. Nevertheless, the proposed technique has the potential to dramatically increase the IoMT system’s security and dependability while simultaneously minimizing threat likelihood.

To further support our evaluations, we carry out a *t*-test (note that other hypothesis testing can be carried out) to test the *null hypothesis* whether the proposed framework and literature works would provide the same level of protection to the IoMT systems. Various statistics are given in Table 6, such as *t Stat*, *t Critical two-tail*, and the *Mean Differences*. It can be observed that *t Stat* values compared to *t Critical two-tail*, for the proposed framework, are consistently higher than the previously published works. As an example, examining the first column i.e., comparing proposed framework with Alrawashdeh, et al. [18], the tStat>tCriticaltwo−tail are evaluated to 3.55>2.57, hence we reject the null hypothesis, whereas carrying a huge *mean difference* of 0.15. Similarly, for some other proposals e.g., [19,20,24], there is a huge difference among various statistics, which is convincing enough to claim that the security provision with the proposed approach is significantly different from these proposals. Similar trends can also be observed with other proposals i.e., [25,27,28].

## 6. Conclusions

Large or small, regardless of size, each institute is vulnerable to devastating effects of ransomware attack. Cyber criminals have been developing vulnerabilities at a more rapid pace than system developers have programmed protections in several recent incidents. The risk associated with ransomware begins with a preliminary infection and compromise, but it does not end there. In order to avoid vulnerability, a defense system should be autonomic and autonomous. The proposed technique leveraged multiple capabilities, such as monitoring, identification and alerting of abnormal sourcing patterns for incident response. Vulnerability analysis enabled the ransomware prevention system to respond in an active manner. During the experimental assessment, the proposed methodology was reviewed and trained to achieve resiliency with integrated recovery skills. The malware prevention shield limits the ability of the adversary to create highly targeted attacks. To eliminate blind spots, in future we aim to focus on:

1. Explore opportunities to bridge security monitoring gaps.
2. Examine the impact of packet traffic monitoring to purpose secure hybrid environment (i.e., on-premises and in the cloud) that can operate without friction,
3. Indicate appropriate methodology to gain scalable system audit log in order to gain valuable insights.
4. Detect next-generation and emerging ransomware threats in real-time with higher efficiency and diminished false positives.

## Figures and Tables

**Table 1:** Assessment of similarities and differences of Related Work.

Research	Narrative	Progression	Covered Features of Ransomware				Covered Platforms		
			Objectives	Contagion	Malevolent Events	Extortion	PCs	Mobile Devices	IoT
Alrawashdeh et al. [18]	To effectively detect malicious activity, used deep learning to extract the latent representation of high-dimensional data.	N	I	I	I	N	I	I	F
Homayoun et al. [19]	Reveals IoT Ransomware development.	I	I	I	F	I	N	I	F
Azmoodeh et al. [20]	Demonstrated an Android power consumption-based machine learning method to identify ransomware assaults.	I	F	I	I	N	N	F	F
Fernandez et al. [24]	Examined how genetic and nature-inspired attribute selection methods work in systems with unpredictable forecasting modifications.	I	I	I	I	N	N	N	I
Damien et al. [27]	Examined how evolutionary and nature-inspired attribute selection techniques work in settings where the forecasting model evolves unexpectedly.	I	I	I	I	N	I	I	I
Umme et al. [27]	Used Zero-shot Learning (ZSL) to identify ransomware.	I	I	I	I	I	I	I	I
Manabu et al. [28]	Presented a novel dataset of ransomware configurations on several operating systems and storage system with complete ciphering.	F	I	I	F	I	I	I	I

N = No details are furnished, I = Incomplete data furnished, F = Full details are furnished.

**Table 2:** System Service States.

State	Description	Auto-Restart	On-Boot	On-Reboot	Post Package Installation	Pre Package Updates
Ready	Application has begun (i.e., setting up the dbus connection)	No	No	Not auto launched when reboot	Not auto-launched callback (i.e., service_app) when reboot	Not launched inevitably
Created	Application initiates the core loop.	No	Yes	Launched automatically	Initiated callback (i.e., service_app)	Launched inevitably
Running	Application is functional.	Yes	No	Not Launched	Not launched the callback (i.e., service_app)	Launched inevitably
Terminated	Application’s functional operation has ended	Yes	Yes	Not Launched	Launched callback (i.e., service_app)	Launched inevitably

**Table 3:** Analyzed malware that was used in experiments.

			Attack Types		
**Malware**	**Samples**	**Variant**	**Encrypting Files**	**Deleting Files**	**Stealing Files**
GPcode	55	9	Yes	No	No
Filecoder	46	5	Yes	Yes	No
Cryptolocker	30	5	Yes	No	Yes
Tobfy (screen locker)	15	4	Yes	No	Yes
Cryptowall	11	3	Yes	No	No
AutoKMS	9	6	No	Yes	Yes
Wacatac.B!ml	11	4	Yes	Yes	Yes
Obfuscator	7	5	Yes	No	No
Daemon_Tools_Lite_BundleInstaller	10	5	Yes	Yes	Yes

**Table 4:** Observed attributes for malicious software and/or process call.

Operation	Purpose
DLL removable execution	Hijack Execution Flow
Unwanted Executable Image	Exploit targets
Outdated Application Version	Inject executable malicious code
Size of Executable Code	Identify and/or payload exploitation
Size of Reserved Stack	Off-by-one overflow indicator
Size of Heap Stack	Off-by-one overflow indicator
Size of Reserved Commit	Log4Shell exploitation
Size of Heap Commit	Heap-based buffer overflow
Address of Entry Point	Understanding Privilege Escalation
Data Source	Understanding Privilege Escalation, Command Injection Attacks
Application Loader Flags	Identification of Code Flaws, and Insecure Code
Data Relocation Calls	Identify attack vectors
Pointer to Raw	Identify attack vectors
Data Applied Primary & Sub Languages by Application	Identify ‘System call parameters and application ‘Configuration settings’
Timestamp of Executable Code Generation	Exploit DB to launch Denial-of-Service

**Table 5:** Configuration for experimental evaluation.

Operating System	Tizen 6.0 M2
OS Kernel	Linux LTS with multiuser support
File format	Tizen Package Kit (TPK)
De-compilation Tool	dnSPY
CPU	AMD A10-4600M, AMD A6-3400M
Data Width	64 bits
L1 Data	4 * 64 Kbytes (2-way)
CPU Cores & Threads	4
Frequency	1400 to 2300 MHz
Bus Speed	99.82 MHz
Thermal Power	35 Watt
Power state levels	Screen (OFF, DIM, NORMAL, AWAKE)
RAM	8 GB
Devices	X86 supportive architecture (wearables, mobile, etc.)
Transistor Count	1303 million
Total Data/Code Samples	194
Analyzed Ciphered File Extensions	.FOX, .KOKO8, FASTB, .ANN
Targeted Extensions	.txt, .doc, .docx, .xls, .xlsx, .jpg, jpeg, .mdb, .zip, .rar
Data Mining Tool (Testing and Validation)	KNIME (Konstanz Information Miner)

**Table 6:** Statistical hypothesis testing for proposed framework and previously published works.

Statistical Symbols	Proposed Research						
t Stat	3.55	11.49	12.82	15.64	8.06	9.74	6.67
t Critical two-tail	2.57	2.31	2.31	2.31	2.36	2.36	2.45
Mean Differences	0.15	0.26	0.25	0.36	0.19	0.19	0.20
	[18]	[19]	[20]	[24]	[25]	[27]	[28]

## Data Availability

Not applicable.
